# Ecology and Biology of Aquatic Insects

**DOI:** 10.3390/insects12010051

**Published:** 2021-01-11

**Authors:** Scott M. Starr, John R. Wallace

**Affiliations:** 1 Department of Plant and Soil Sciences, Texas Tech University, Lubbock, TX 79409, USA; scott.starr@ttu.edu; 2 Department of Biology, Millersville University, Millersville, PA 17551, USA

## 1. Introduction

The advancement of our knowledge on the ecology and biology of aquatic insects is essential to improving our understanding of their roles in water quality, disease ecology, as indicators of climate change, biodiversity, as well as community structure and ecosystem functioning. Over the past 100 years, large strides in research have been made in the ecology and biology of aquatic insects that have expanded our knowledge on their diversity, life histories, potential as surrogates for ecosystem attributes, as well as ecosystem energetics [1]. Aquatic insects are found within the interfaces of terrestrial and mainly freshwater ecosystems such as lentic systems, e.g., lakes, ponds, wetlands, bogs, as well as lotic systems, e.g., springs, streams, and rivers, while only a few occur in truly marine habitats. Aquatic insect communities can vary greatly within and among habitats but also according to how humans have altered adjacent lands, and these communities play significant roles within the freshwater ecosystems they inhabit whether through the cycling of nutrients or via their overall contribution to secondary production. Aquatic insects contribute to the trophic structure of the ecosystems by filling functional roles ranging from detritivores up to predators, along with being food sources for vertebrate and invertebrate predators. As many aquatic insects have both aquatic (larval and adult) and terrestrial (adult) life stages, their impact is not limited to the aquatic environment alone and stretches into the terrestrial riparian environment. This “Ecology and Biology of Aquatic Insects” Special Issue will address current basic and applied areas of research focused on aquatic insect evolution, habitat partitioning, community response to land use/land change relative to disease ecology, their application as surrogates for ecosystem attributes, such as with human-mediated drivers of pollution, as well as their use in the court of law.

A major process that has presented an essential problem for insects to be evolutionarily successful in their adoption of aquatic habitats is respiration [2]. Insects have resolved this issue in many different ways, a few of which include cutaneous and gill respiration and through the extraction of air from plants. Field ecological studies and molecular techniques can be used to not only better understand how these types of physiological mechanisms have allowed unique ways for aquatic insects to respire, but also understand how they have adapted to flowing waters as well as developed control strategies for pest species. This issue will address how transcriptomic analyses can be used to control a pest species of aquatic beetle.

Currently, aquatic insects are comprised of more than 88,500 described species from approximately 13 orders [2,3]. Although this significant relationship between taxonomy and basic biological and ecological studies has been widely recognized, major groups of aquatic insects remain poorly understood, e.g., Plecoptera, Trichoptera, and even less so with minor groups such as Neuroptera. Many of these species are understudied, leaving gaps in ecological knowledge regarding habitat use or partitioning about either the larval, adult, or both life stages, e.g., the aquatic Neuropteran family, Sisyridae (Spongillaflies). A lack of species-level information may hamper our understanding of aquatic insect life histories and their role in production and bioenergetics [2]. This issue will include studies that address habitat use and partitioning by two of these orders in an attempt to augment our ecological knowledge of different life stage and life history attributes.

Aquatic insect communities can be found on a gradient—from very biologically heterogeneous to very homogeneous, in diversity and abundance—that is influenced by environmental factors. These organisms require specific hydraulic, substrate and oxygen requirements that can cause spatially distinct communities [4]. Aquatic insect community diversity and integrity are important due to the functional ecosystem roles they perform; therefore, landscape, biotic, abiotic, and anthropogenic factors can all influence the community composition. This issue will address how certain insect community composition responds to stream flow and anthropogenic impacts as drivers of community composition in disease ecology.

Changes in aquatic ecosystems, whether naturally or anthropogenically caused, can result in changes in relative proportions of specific aquatic insect groups [1]. Aquatic insects are often used as surrogates to assess water quality and pollution impacts in freshwater environments. For example, the EPT (Ephemeroptera, Trichoptera, and Plecoptera) richness index is one example of surrogacy that is widely used in many aquatic studies. The richness of these three pollution intolerant orders is used as a metric for overall environment health, water quality, and response to pollution in many research studies [5]. This issue will address how surrogates for ecosystem attributes are being used in the emerging human-mediated pollution of microplastics.

The application of aquatic insect ecology and their biology has been intertwined in various aspects of human society, such as in art, music, a source for food and for recreational sports such as fly-fishing. An emerging application to the use of aquatic insects in human society is how they can be utilized in the court of law, specifically with death scene investigations [6,7]. This issue will evaluate the use of aquatic Chironomidae in death investigations and how this group of aquatic insects may provide valuable details in estimating a postmortem submersion interval (PMSI).

This Special Issue on the Ecology and Biology of Aquatic Insects brings new empirical results and aids in unraveling aquatic insects’ complex roles in the aquatic community and to ecosystem function.

## References

[1] Cummins K.W., Merritt R.W., Berg M.B., Merritt R.W., Cummins K.W., Berg M.B. (2019). Ecology and distribution of aquatic insects. An Introduction of Aquatic Insects of North America.

[2] Huryn A.D., Wallace J.B., Merritt R.W., Cummins K.W., Berg M.B. (2019). Habitat, life history, secondary production, and behavioral adaptations of aquatic insects. An Introduction of Aquatic Insects of North America.

[3] Dijkstra K.D.B., Monaghan M.T., Pauls S.U. (2014). Freshwater biodiversity and insect diversification. Annu. Rev. Entomol..

[4] Starr S.M., Benstead J.P., Sponseller R.A. (2014). Spatial and temporal organization of macroinvertebrate assemblages in a lowland floodplain ecosystem. Landsc. Ecol..

[5] Herman M.R., Nejadhashemi A.P. (2015). A review of macroinvertebrate and fish-based stream health indices. Ecohydrol. Hydrobiol..

[6] Wallace J.R., Merritt R.W., Byrd J.H., Tomberlin J.K. (2020). Chapter 7: The role of aquatic organisms in forensic investigations. The Utility of Arthropods in Legal Investigations.

[7] Wallace J.R., Merritt R.W., Kimbirauskas R., Benbow M.E., McIntosh M. (2008). Caddisfly cases assist homicide case: Determining a postmortem submersion interval (PMSI) using aquatic insects. J. Forensic Sci..

